# Metals and Extremophilic Bacteria in Mining Environments: A Systematic Review

**DOI:** 10.3390/microorganisms14061312

**Published:** 2026-06-11

**Authors:** Joseline Jiménez-Venegas, Leonardo Zamora-Leiva, Celián Román-Figueroa, Yasna Tapia, Manuel Paneque

**Affiliations:** 1 Faculty of Agricultural Sciences, University of Chile, Santa Rosa 11315, La Pintana, Santiago 8820808, Chile; jjimenezve@fen.uchile.cl (J.J.-V.); yasnatapiafernandez@uchile.cl (Y.T.); 2 Master Program in Territorial Management of Natural Resources, University of Chile, Santa Rosa 11315, La Pintana, Santiago 8820808, Chile; 3 Bionostra Chile Research Foundation, Almirante Lynch 1179, San Miguel, Santiago 8920033, Chile; lzamora@bionostra.com (L.Z.-L.); croman@bionostra.com (C.R.-F.)

**Keywords:** mining tailings, bioremediation, biotechnology

## Abstract

Industrial activities have contributed substantially to the global economy but have also resulted in the release of hazardous substances into the environment. This systematic review aimed to identify extremophilic or extremotolerant bacteria capable of surviving high metal concentrations and actively remediating elevated levels of Cd, Cr, Cu, Fe, Pb, and Zn. Following the PRISMA guidelines, a qualitative systematic review was conducted in the Web of Science and Scopus databases for studies published between 2000 and 2025 (last search: 5 January 2026). The synthesized dataset revealed distinct ecological and functional roles across different taxonomic levels. At the family level, Carnobacteriaceae, Cyclobacteriaceae, and Erythrobacteraceae were predominantly associated with high metal tolerance (“exposed” profiles) in alkaline environments. Conversely, at the genus level, *Acidithiobacillus*, *Phenobacterium*, *Microbulbifer*, and *Roseobacter* demonstrated high active remediation capacities in acidic settings through bioleaching, precipitation, or biosorption. Species such as *Bacillus subtilis* and *Acidithiobacillus ferrooxidans* exhibit a dual profile combining environmental tolerance and high bioremediation performance. These findings highlight methodologically heterogeneous studies, necessitating standardized experimental validation prior to large-scale technological deployment.

## 1. Introduction

Extremophilic microorganisms can survive a wide range of extreme conditions, including high or low temperatures [1,2], high salt content [3,4], acidic or alkaline pH [5], high metal concentrations [6], high pressure [7], and elevated radiation levels [8]. Their physiological and metabolic adaptations have garnered interest because of their potential use as biotechnological tools for the remediation of contaminated environments [9].

Bacteria (including cyanobacteria and actinomycetes), fungi, and algae [10,11,12,13,14] can thrive in metal-rich environments, using metals as essential components of cellular processes [15,16]. Bacteria from the genera *Bacillus*, *Pseudomonas*, *Actinomyces*, *Serratia*, *Enterobacter*, *Thiobacillus*, *Rhodobacter*, *Agrobacterium*, and *Acidithiobacillus*, among others [17,18,19], have been reported to tolerate, bioaccumulate, and biotransform metals through mechanisms such as biosorption, precipitation, and chelation [20,21,22,23]. These capabilities enable their use as bioindicators of metal contamination and support their application in ex situ and in situ ecosystem restoration strategies [15,24,25]. Notably, *Acidithiobacillus* species play a dual environmental role. Although widely applied in controlled bioleaching processes, their sulfur- and iron-oxidizing metabolism can also promote the formation of acid mine drainage (AMD) in unmanaged mine waste, increasing acidity and metal mobilization [26]. Importantly, these processes are typically mediated by complex microbial consortia, in which *Acidithiobacillus* species are commonly present but do not act in isolation. This ecological context highlights the need to evaluate remediation potential in a context-dependent manner [27].

Extremophilic microorganisms have been reported in a wide variety of environments, ranging from soils and rice paddies with metal presence to salt flats, desert systems [28,29,30,31,32], aquatic or volcanic environments, and even alkaline ashes [33,34,35]. Unlike other contaminated environments, contaminated mining sites are characterized by the coexistence of high metal concentrations, extreme pH values, and potentially toxic compounds originating from extractive activities [36]. These harsh conditions restrict conventional remediation approaches while simultaneously selecting microorganisms capable of surviving under such stress. The presence of microorganisms under these extreme conditions highlights the adaptive potential of these extremophiles. Their metabolic and enzymatic capabilities enable processes such as the biosorption, precipitation, and biotransformation of metals, making them promising candidates for bioremediation strategies [37,38].

Previous reviews have addressed environmental remediation at mining sites [39,40,41]; however, they primarily focused on tailings characterization, management of mining liabilities, or general remediation strategies, without systematically integrating the diversity of extremophilic or extremotolerant bacteria or their specific mechanisms for tolerating and remediating multiple metals under extreme pH and high-metal-concentration conditions [42,43]. These limitations hinder our understanding of their biotechnological potential and practical applications in environmental rehabilitation [37,38], as knowledge of their interactions with the ecosystem, remediation effectiveness against metal mixtures, and optimal conditions for maximizing performance in real mining environments remains limited [44,45]. While restoration strategies in metal-polluted soils have mainly focused on phytoremediation [44], studies addressing microbial communities and their functional roles in complex metal environments remain scarce [45].

In this review, we aimed to identify extremophilic or extremotolerant bacteria with the potential to remediate environments containing high concentrations of heavy metals. These microorganisms are particularly relevant in mining tailings, where extreme environmental conditions and contamination levels present challenges that conventional microorganisms cannot overcome [43]. This review broadens our current understanding of bacterial tolerance and remediation mechanisms, contributes to the development of targeted strategies for the effective and sustainable mitigation of mining-related environmental impacts, and fills current knowledge gaps by providing a systematic and up-to-date analysis of extremophilic or extremotolerant bacteria with remediation potential, highlighting their mechanisms of action and relevance for developing sustainable environmental restoration strategies in mining-impacted ecosystems.

## 2. Materials and Methods

### 2.1. Systematic Review Protocol

Information on extremophilic or extremotolerant bacteria for heavy metal remediation was systematically sourced from electronic scientific databases, such as Scopus and Clarivate Analytics’ Web of Science. This systematic review was conducted according to the Preferred Reporting Items for Systematic Reviews and Meta-Analyses (PRISMA) 2020 guidelines [46]. Systematic searches were conducted on 5 January 2026. This study was not registered in PROSPERO because it does not fall within the scope eligible for registration.

The first search focused on extremophilic or extremotolerant bacteria associated with environments containing high metal concentrations. The search string used was *Extremophilic microorganisms*: “microorganisms AND extremophile AND (heavy OR trace) AND metal”. The second search aimed to identify bacteria with the potential to remediate metal contamination. The search string used was *Remediation bacteria*: “(metal OR metals) AND remediation AND microorganism AND contamination”.

**Figure 1:**
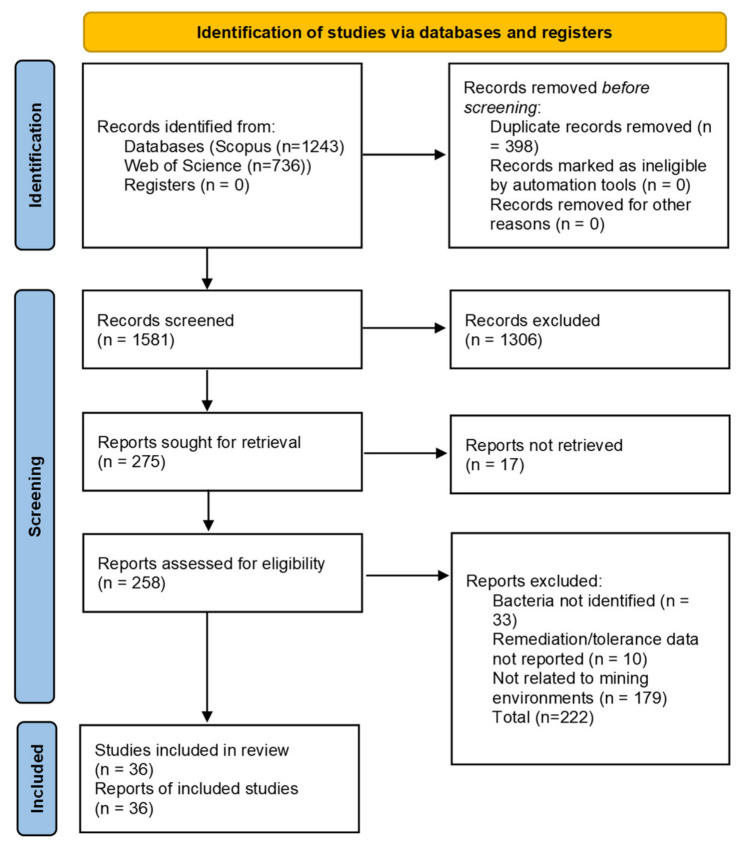
PRISMA 2020 flow diagram illustrating the study selection process for the systematic review of extremophilic and metal-remediating bacteria. Adapted from the PRISMA 2020 statement.

The study selection process is illustrated in the PRISMA flow diagram (Figure 1).

### 2.2. Article Selection and Eligibility

Records retrieved from each search were processed independently, and duplicate entries from each dataset were eliminated. The review included publications from 2000 to 2025, and only peer-reviewed records written in English or Spanish were considered. The inclusion and exclusion criteria are listed in Table 1.

Non-mining environments were excluded to ensure that the review focused on systems characterized by extreme pH values, high metal loads, and mineral-rich matrices, typical of mining environments [47]. These geochemical conditions impose strong selective pressures that shape unique microbial tolerance and remediation mechanisms, making mining contexts the most relevant setting for addressing the aims of this review. A single reviewer (J.J.-V.) performed an initial screening to ensure consistency. To minimize subjectivity, borderline cases were reassessed and discussed with second (L.Z.-L.) and third (C.R.-F.) reviewers.

### 2.3. Data Extraction and Analysis

All relevant data were extracted from the eligible studies, including taxonomic identification (species, genus, or family), environmental context of bacterial development, reported metal concentrations (exposed and/or remediated), mechanisms of remediation, and associations with plant species (if applicable). Data extraction, application of the eligibility filters, and construction of evidence exraction and risk of bias matrices were managed using Microsoft Excel 365 (Microsoft Corporation, Redmond, WA, USA). This information was systematically tabulated and analyzed. For the purposes of this review, metal–bacteria interactions were classified into two categories based on two independent systematic searches: (i) studies reporting extremophilic or metal-tolerant bacteria in environments with high metal concentrations, and (ii) studies evaluating bacteria with experimentally demonstrated metal remediation capacity in mining-related environments. In the first category, the term exposed was used to describe studies in which bacteria were reported to inhabit or persist in environments with measured metal concentrations, but without experimental evidence of an active remediation mechanism. In the second category, terms such as bioaccumulation, biosorption, precipitation, immobilization, or bioleaching were used only when the article explicitly evaluated a metal-removal process. Prior to synthesis, the extracted data were standardized to facilitate comparisons among the studies. When possible, the reported metal concentrations were converted to mg/kg to ensure consistency across datasets. In addition, bacterial names were reviewed and harmonized according to the current taxonomic nomenclature to maintain a uniform format throughout the analysis.

After eligible articles from both searches were processed independently, a cross-referencing step was performed to identify bacterial taxa present in both datasets. This process involved manually matching species, genera, and families reported in studies (i) describing extremophilic metal-tolerant bacteria and (ii) evaluating bacterial metal remediation. Taxa appearing in both datasets were classified as jointly relevant because they combined physiological tolerance to metal-rich extreme environments with experimentally demonstrated remediation capacity. These cross-referenced taxa are subsequently highlighted in the Section 3.3 and Section 3.4  to integrate both lines of evidence.

A qualitative synthesis was conducted to compare the reported extremophilic microorganisms and metal-remediating bacteria in the literature. Owing to the heterogeneity of the study designs and reported variables, a quantitative meta-analysis was not performed.

The extracted data were organized into summary tables to present the bacterial taxa, environmental conditions, and reported metal tolerance or remediation capacities. Visual summaries were prepared to illustrate the relationships between extremophilic microorganisms and metal-remediating bacteria.

### 2.4. Quality and Risk of Bias Assessment

To guarantee the reliability, reproducibility, and internal validity of the synthesized evidence, a formal risk of bias assessment was performed for all included studies. Given the non-randomized, observational, and experimental nature of environmental microbiology literature, the standard Cochrane tools for clinical trials were not applicable.

According to the guidelines of the Collaboration for Environmental Evidence (CEE), evidence synthesis in environmental management requires customized critical appraisal tools because the core units of analysis are experimental laboratory setups rather than clinical human cohorts; failing to assess these specific technical variations can lead to inaccurate or skewed systematic conclusions [48]. Furthermore, as emphasized by Kohl et al. (2015) [49] for biological and bioengineering evidence assessments, when predefined standard tools for specialized laboratory designs are lacking, authors must establish an ad hoc tailored strategy focused explicitly on internal validity and methodological rigor.

Consequently, behavioral human-centric bias domains (e.g., deviations from intended medical interventions or patient dropouts) were excluded because of their lack of biological relevance in benchtop or field microbial research. Instead, an ad hoc tailored checklist was developed by adapting the core structural logic of the Risk of Bias in Non-randomized Studies of Interventions (ROBINS-I) and Exposure (ROBINS-E) frameworks, following environmental and public health adaptation methodologies [50,51,52]. This domain-based adaptation aligns with recent precedents in environmental synthesis, where quality appraisal criteria are custom-tailored to the specific experimental parameters of microbial bioremediation [53].

To prevent methodological confounding and respect the bimodal design of this review, two independent evaluation matrices were established with distinct domain criteria tailored to the specific objectives of each systematic dataset.

#### 2.4.1. Exposure and Tolerance Studies Checklist (20 Articles): Focused on Capturing Analytical and Ecological Rigor, Evaluating Five Specific Domains

• (D1) Selection and environmental characterization of sampling sites.
• (D2) Measurement of metal exposure using high-precision instrumental quantification (e.g., ICP-MS, ICP-OES, or ASS) to avoid nominal dosing bias.
• (D3) Robust molecular and taxonomic identification of extremophile isolates (e.g., 16S rRNA gene sequencing with public repository accession numbers).
• (D4) Control of operational and environmental confounding factors (e.g., temperature and buffered initial pH).
• (D5) Selective reporting of results across all tested strains and metals.

#### 2.4.2. Experimental Remediation Studies Checklist (16 Articles): Focused on the Internal Validity and Experimental Design Controls of Active In Vitro or In Situ Removal Assays, Evaluating Five Domains

• (D1) Experimental design validity and the mandatory inclusion of parallel sterile abiotic controls to differentiate microbially mediated removal from spontaneous chemical precipitation.
• (D2) Strict adherence to and reporting of protocol conditions (e.g., incubation time, agitation speed, and mass balance validation).
• (D3) Taxonomic traceability, purity validation, and biological characterization of the remediating strain or complex microbial consortium.
• (D4) Active monitoring and control of chemical confounding factors (e.g., kinetic fluctuations in pH capable of inducing spontaneous chemical precipitation).
• (D5) Reporting of experimental replicates (minimum in triplicates) along with statistical dispersion parameters.

Following the established ROBINS-I domain-based logic [54], the risk of bias was not quantified using arbitrary numerical scales or cumulative scoring systems, as mathematical weights can obscure critical methodological flaws [55,56]. Instead, a qualitative, rule-based approach was implemented. Each domain was independently classified as low risk (+), moderate/unclear (?), or high risk (−).

The Overall Risk of Bias for each study was determined using a restrictive threshold: a study was categorized as “low risk” only if all domains achieved low risk; “moderate risk” if it presented unclear compliance in one or more domains but lacked critical failures; and “high risk” if it exhibited an explicit high risk or total absence of control in at least one fundamental domain (such as the omission of abiotic controls or lack of experimental replicates), which inherently compromises the validity of the entire biological outcome.

## 3. Results

A total of 1979 records were retrieved from the systematic searches. After removing duplicates and applying the eligibility criteria, 36 studies were included: 20 reported extremophilic metal-tolerant bacteria, and 16 reported metal-remediating bacteria. The study selection process is illustrated in the PRISMA flow diagram (Figure 1). A total of 17 articles were excluded due to lack of access to the full text; among these, 12 appeared to meet the inclusion criteria based on their title and abstract but could not be fully assessed and were therefore excluded from the final analysis.

### 3.1. Identification of Metallotolerant Bacteria

In the initial search for extremophilic microorganisms, 164 articles were retrieved: 50 from the Scopus database and 114 from the Web of Science database. Of these, 144 were unique records. Furthermore, 124 articles did not meet the inclusion criteria for the following reasons: 4 were book chapters, 1 was a book, 1 was a conference paper, 1 was a note, 3 were proceeding papers, 39 were reviews, 23 did not report metal data, 6 were not about extremophiles, 33 did not identify bacteria, and 13 were inaccessible. Ultimately, 20 articles were included in the qualitative synthesis (Table 2). A detailed summary of the environmental metal concentrations reported in these studies and the corresponding extremophilic or extremotolerant bacteria identified are presented in Table 2.

Articles identified in this search showed a concentration within the last 5 years, with the oldest dating back to 2006. The metals most frequently reported were Cd, Cr, Cu, Fe, Pb, and Zn, with the study by Ramanathan and Ting [34] evaluating the highest number of these elements (Cd, Cr, Cu, Fe, Pb, and Zn).

In the search for remediation bacteria, 1815 articles were identified: 1193 from Scopus and 623 from the Web of Science database. A total of 378 duplicate records were removed, and 1421 articles were excluded for the following reasons: 44 due to language, 5 books, 189 book chapters, 24 conference papers, 2 editorials, 1 note, 12 proceedings papers, 2 retracted articles, 410 reviews, 2 short surveys, 326 unrelated to microorganisms, 131 unrelated to remediation, 83 that did not report metals, 7 that did not report remediation amounts, 179 unrelated to mining environments, and 4 that were inaccessible. Ultimately, 16 articles were included in the qualitative synthesis (Table 3).

Table 3 summarizes the studies that focused on bacterial metal remediation in mining-related environments, representing the second independent dataset generated in this study. Together with the extremophilic dataset (Table 2), these results were subsequently used for cross-referencing to identify bacterial taxa exhibiting both metal tolerance and remediation capacity.

Most articles were published within the last 5 years, with the oldest dating back to 2001. As in the first search, the metals most frequently reported were Cd, Cr, Cu, Fe, Pb, and Zn. However, the remediation of at least four metals (Cd, Cu, Pb, and Zn) was reported only by Liu et al. [78].

### 3.2. Methodological Quality and Risk of Bias Assessment

**Figure 2:**
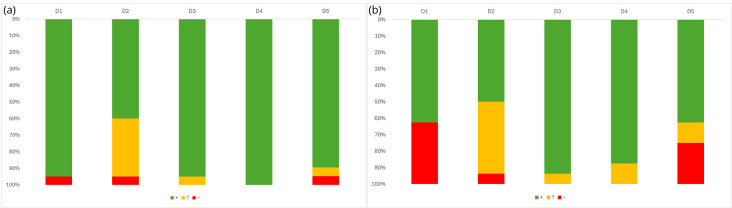
Methodological quality and risk of bias assessment of the included literature. (**a**) Evaluation of studies focusing on heavy metal tolerance mechanisms (*n* = 20). (**b**) Evaluation of studies focusing on bioremediation and heavy metal removal potential (*n* = 16). Green, yellow, and red bars represent low risk (+), moderate risk (?), and high risk (−) of bias, respectively, across the five experimental domains (D1–D5). Detailed study-by-study justifications are provided in Tables S1 and S2 in the Supplementary Materials.

The methodological rigor and risk of bias of the selected studies were systematically evaluated across five experimental domains (D1 to D5). Due to the distinct experimental nature of the reviewed literature, the assessment was divided into two core categories: heavy metal tolerance mechanisms (*n* = 20 studies), and bioremediation or removal potential (*n* = 16 studies) (Figure 2).

For the studies investigating heavy metal tolerance mechanisms, outstanding methodological compliance was observed in Domain 4 (Control of Confounders), where 100% of the reviewed papers achieved a low risk of bias profile (Figure 2a). This indicates robust and consistent standardization across the literature regarding critical experimental parameters prior to metal exposure. High compliance was also verified in Domain 1 (Selection and Baseline Controls) and Domain 3 (Outcome Measurement), with 95% of the studies meeting all quality criteria in both domains, while a minor 5% presented a moderate risk. These results demonstrate that most tolerance investigations provide precise characterizations of the diverse source environments of the strains (contaminated sites, extreme environments, salt lakes, or deserts) and rigorous tracking of alterations in bacterial growth curves under heavy metal stress.

However, significant methodological gaps were identified in Domain 2 (Experimental Design and Timeline), where 35% of the studies were classified as moderate risk and 5% as high risk. This penalization was primarily driven by the nominal dosing of metals (theoretical calculations lacking instrumental analytical verification of the actual initial concentration in the medium) and specific deficiencies in the inoculum reporting. Additionally, Domain 5 (Reporting and Replicates) showed that 10% of the studies presented a high risk due to the omission of independent biological replicates in their kinetic assays. In the overall synthesis (General Domain) of the tolerance literature, 75% of the studies were categorized as low risk, 5% as moderate risk, and 20% as high risk.

In contrast, the evaluation of studies focusing on bioremediation and removal potential––characterized by their direct focus on the reclamation of mining environments, industrial effluents, and applied biotechnological applications––revealed substantial challenges in experimental design (Figure 2b). While Domain 3 (Outcome Measurement) demonstrated excellent adherence, with 93.8% of the studies utilizing appropriate analytical instrumentation (such as ICP-AES or ICP-OES) to measure residual removal, severe limitations were identified in Domain 1 (Selection and Baseline Controls) and Domain 2 (Experimental Design). Notably, 37.5% of the removal studies were classified as having a high risk of bias in D1 because of the complete lack of sterile abiotic controls. This omission is critical in the context of the remediation of mining soils, leachates, and effluents, as it precludes the ability to differentiate truly microbially mediated processes (such as bioleaching, bioaccumulation, or induced precipitation) from spontaneous chemical precipitation or physical adsorption within the analyzed matrices (e.g., soil columns or lysimeters).

Furthermore, 43.8% of these remediation assays exhibited a moderate risk in Domain 2 due to failures in accurately standardizing the initial density of the inoculated biomass. Domain 5 also evidenced deficiencies, with 25% of the studies at high risk for reporting removal rates as single absolute values without the minimum statistical replicates (*n* ≥ 3). When considering the overall quality (General Domain) for the remediation studies, 50% achieved a low-risk profile, while 12.5% were classified as moderate risk, and 37.5% were identified as having a high methodological risk. Detailed, study-by-study justifications for each domain assignment are extensively documented in Tables S1 and S2 in the Supplementary Materials.

### 3.3. Performance and Operational Ranges of Active Metal-Remediating Bacteria

The values compiled in Table 4 correspond exclusively to the outcomes of the systematic dataset that evaluated active microbial bioremediation. Unlike the baseline survival metrics, all terms listed in the “*Metal interaction*” column denote experimentally demonstrated metal removal, immobilization, or bioleaching processes, and the associated values represent measured active remediation concentrations. While the baseline environmental metal exposure levels and ecological tolerance profiles for these extremophilic and extremotolerant strains are comprehensively detailed within the raw study extraction data in the Supplementary Materials (Tables S1 and S2), Table 4 highlights their functional performance. This streamlined dataset allows for the direct identification of high-potential taxa that combine extreme environmental resilience with validated active remediation capacities by cross-referencing the text findings with supplementary records.

### 3.4. Analysis of Metallotolerant Bacteria

Bacillaceae was particularly noticeable among the main families identified in the articles, with 40 species exposed to different metal concentrations. Within this family, the genus *Bacillus* is the most frequently cited, with 16 species reported to tolerate a wide range of pH conditions, from acidic (pH 3.0) [81] to alkaline (pH 11.98) [34] conditions. However, in some cases, this tolerance may be associated with survival strategies, such as endospore formation, which allows persistence under extreme conditions without necessarily implying active metabolic growth. Additionally, the Carnobacteriaceae family included three species exposed to various metal concentrations under alkaline conditions (pH 10.55) [34] (Table S3). Among the families reported to have metal remediation capacity, several taxa were associated with Cu removal under acidic conditions, with reported concentrations reaching up to 816 mg/kg [79] (Table 4). These studies also indicated that some of these bacteria are able to persist across a broader pH range, including near-neutral environments.

Bacteria reported under metal exposure conditions were identified in both soil and aquatic systems; however, soil environments were more frequently represented (7 studies) than aquatic systems (5 studies), with an additional group of studies conducted under experimental or non-environment-specific conditions. All studies reported high metal concentrations. In soil environments, a wide range of conditions have been described, including desert soils from the Atacama Desert [31], Antarctic soils [58], mining soils [60], industrial soils [59,62], agricultural soils [64], and Antarctic marine sediments [63]. In aquatic environments, studies have mainly focused on extreme or contaminated systems, such as AMD [57], saline lakes [29], river sediments [65], and hypersaline or alkaline lake systems [66], as well as experimental aqueous systems for metal removal [35].

Studies on bacterial remediation have been conducted across both soil and aquatic systems, with a predominance of soil-based environments, particularly those associated with mining activities, such as contaminated soils, tailings, and adjacent agricultural areas impacted by mining [67,68,69,72,73,75,76,78,82,84]. These environments are frequently associated with bacterial taxa capable of metal removal through mechanisms such as biosorption, precipitation, and bioleaching. In aquatic systems, research has mainly addressed mining-related waters, particularly AMD, coal mine drainage, and tailing effluents, as well as engineered systems that treat metal-contaminated water [61,70,71,74,77,81]. These systems are commonly associated with bacteria involved in metal mobilization or removal processes under acidic to near-neutral conditions. Although the distribution of studies reflects a greater research focus on soil environments (50%) than on aquatic systems (31%), the key findings of this review highlight the diversity of bacterial taxa and mechanisms involved in metal remediation across both types of ecosystems.

Pb was the most frequently reported metal (12 articles), followed by Cu (11 articles) and Cd (10 articles), while Cr and Zn were reported in nine articles each. In contrast, Fe was markedly less represented (3 articles). This distribution is consistent with the geochemical profile of mining-impacted environments, where Pb, Cu, and Cd are among the most prevalent and bioavailable metals. Several bacterial families, including Carnobacteriaceae (*A. indicireducens*, *A. pelagium*, *Alkalibacterium* sp.), Bacillaceae (*Bacillus anthracis*), Caryophanaceae (*Bhargavaea cecembensis*), Cyclobacteriaceae (*Fontibacter* sp.), Erythrobacteraceae (*Erythrobacter donghaensis*), and Microbacteriaceae (*Microcella alkaliphila*), were reported to be exposed to concentrations of 610 mg/kg of Cd ([34]; Table S3).

In the bacterial remediation dataset, the reported metal removal capacities varied depending on both the microorganism and environmental conditions. The highest remediation values identified were 643.8 mg/kg Pb [79], 168 mg/kg Zn [73], 63 mg/kg Cr [79], 816 mg/kg Cu [79], 139 mg/kg Cd [77], and 190 mg/kg Fe [70] (Table 4).

The microorganisms associated with these processes include both acidophilic and neutrophilic taxa. Under acidic conditions, *Acidithiobacillus* spp. are primarily associated with bioleaching mechanisms involving Fe, Cu, and Cr. In contrast, under near-neutral pH conditions, *Pseudomonas* spp. and *Bacillus* spp. are associated with biosorption processes, particularly for Cu and Zn. In alkaline environments, Alkaliphilic *Bacillus* spp. are associated with bioaccumulation and precipitation mechanisms involving metals such as Pb, Cd, and Zn.

Notably, a fundamental distinction was made between ecological tolerance and the active biotechnical performance. The term “exposed” refers exclusively to baseline studies in which microorganisms survived under high metal concentrations without quantified evidence of removal. These comprehensive profiles are presented separately in Supplementary Table S3. Conversely, Table 4 compiles taxa with experimentally demonstrated and measured remediation mechanisms (e.g., bioaccumulation, biosorption, or bioleaching) and verified operational pH ranges. This dual-matrix approach prevents methodological confounding while allowing robust cross-referencing between environmental exposure thresholds and active bioremediation performance.

**Figure 3:**
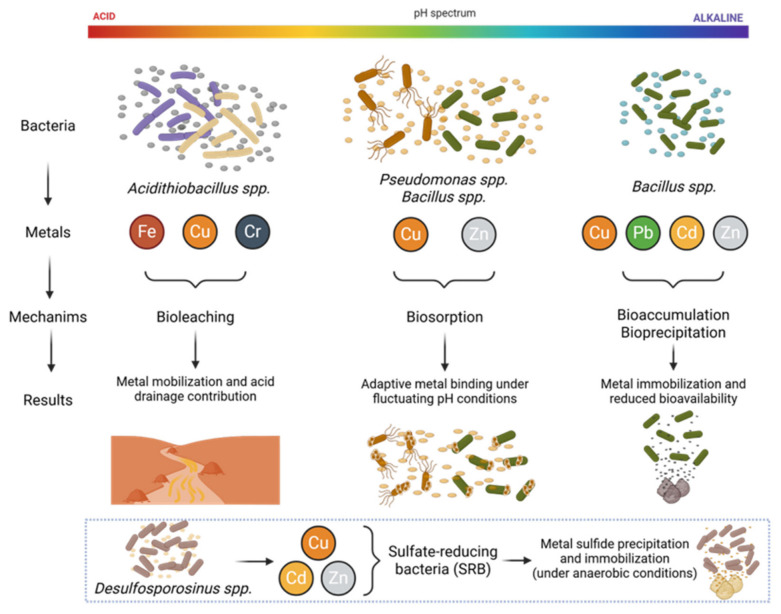
Conceptual overview of bacterial metal remediation mechanisms in mining environments, including pH-dependent and complementary pathways. Acidophilic bacteria (e.g., *Acidithiobacillus* spp.) are primarily associated with bioleaching under acidic conditions, whereas neutrophilic and alkaliphilic bacteria (e.g., *Pseudomonas* spp. and *Bacillus* spp.) contribute to biosorption, bioprecipitation, and immobilization. Additionally, sulfate reduction mediated by sulfate-reducing bacteria (SRB) is a complementary mechanism that occurs under anaerobic conditions, leading to metal sulfide precipitation and immobilization. Although this pathway was less frequently reported in the analyzed dataset, it was included to provide a more comprehensive conceptual framework. Created in BioRender. Bionostra, C. (2026) https://BioRender.com/f7py9mg (accessed on 25 March 2026).

As summarized in Figure 3 and supported by the data presented in Table 4 and Table S3, metal remediation mechanisms varied strongly with environmental pH, with *Acidithiobacillus* spp. promoting Fe, Cu, and Cr bioleaching under acidic conditions, *Pseudomonas* spp. and *Bacillus* spp. performing Cu and Zn biosorption at neutral pH, and alkaliphilic *Bacillus* spp. contributing to the bioaccumulation and bioprecipitation of Cu, Pb, Cd, and Zn under highly alkaline conditions.

Cross-referencing the extremophilic and remediation-focused datasets revealed points of convergence between the two systematic searches. While several genera were identified as metal-tolerant (e.g., *Carnobacterium*, *Erythrobacter*, *Phenobacterium*, and *Microbulbifer*) and others as capable of metal remediation (e.g., *Roseobacter*, *Alkalibacterium*, and *Arthrobacter* [89]), only two species—*Bacillus subtilis* and *Acidithiobacillus ferrooxidans*—consistently appeared in both categories [30,31,57,65,70,73,79,81]. This result emerged directly from the cross-referencing of two independent datasets, rather than from a priori selection.

Although *Bacillus subtilis* is not considered an obligate extremophile, multiple studies have reported extremotolerant strains capable of persisting in highly saline, alkaline, or metal-rich environments, particularly in mining systems. However, within the analyzed dataset, evidence of active metal remediation by *B. subtilis* was limited, with precipitation reported mainly for Pb (up to 495 mg/kg) under specific conditions.

Likewise, the historically used name *Thiobacillus ferrooxidans* corresponds to the currently accepted taxon *Acidithiobacillus ferrooxidans* [85]; here, we adopted the updated nomenclature and retained the former name only when it was cited directly from previous sources. In contrast to *B. subtilis*, *A. ferrooxidans* showed a more consistent association with active metal transformation processes, particularly the bioleaching of Fe, Cu, and Cr under acidic conditions. These taxa were therefore identified as representative species integrating the outcomes of both systematic searches, not because they dominate all remediation mechanisms, but because they uniquely combine evidence of their occurrence in extreme environments with reported interactions with metals in both tolerance and remediation contexts. This distinction highlights the differences in the strength and consistency of the available evidence, with *Acidithiobacillus ferrooxidans* showing a clearer and more consistent role in active metal transformation, whereas *Bacillus subtilis* appears primarily as an extremotolerant species with a more limited documented remediation capacity.

## 4. Discussion

Between 2019 and 2025, recent studies have increasingly focused on metal-tolerant bacteria in mining areas, reflecting a growing interest in understanding the physiological and metabolic strategies that enable microorganisms to persist at high metal concentrations [90]. Mining-impacted environments are relevant systems for studying microbial adaptation to extreme conditions, including high concentrations of heavy metals, extreme pH, and environmental stress [91]. These conditions have been associated with the occurrence of specialized microbial communities exhibiting diverse tolerance and detoxification mechanisms. However, it is important to note that bioprospecting for metal-resistant and metal-remediating microorganisms is not limited to mining environments, as other systems, such as marine and polar ecosystems, have also been widely explored.

In this context, the distribution of metals identified in this review shows that Pb, Cu, and Cd were the most frequently reported elements in both the tolerance and remediation datasets. This pattern is consistent with the geochemical characteristics of mining-impacted environments, where these metals are commonly enriched and bioavailable. The agreement between environmental exposure and experimentally evaluated metals suggests that the current research is largely aligned with the contamination scenarios observed in mine tailings, AMD, and impacted soils.

The results of this study highlighted several bacterial genera associated with metal interactions, including *Acidithiobacillus*, *Pseudomonas*, and *Bacillus*, which can be used for bioleaching [75], immobilization [74,92], or precipitation [81]. These mechanisms are supported by experimental evidence from specific studies (Table 4) and vary depending on the environmental conditions, particularly pH.

### 4.1. Metal-Remediating Bacteria

More than 30 different bacterial species were identified as being exposed to metals across the analyzed studies; however, only a subset of these showed experimentally demonstrated metal-remediation capacities. The following taxa were among the most relevant in the remediation dataset.

*Bacillus subtilis* (CCTCC AB 98002 strain) and *Priestia megaterium* (*B. megaterium* CGMCC 1.223 strain) precipitate Pb under controlled experimental conditions within a pH range of 3–9, with removal values increasing from 250 mg/kg at pH 3 to 495 mg/kg at pH 9 [81,93]. It is important to note that this remediation capacity is specifically associated with Pb and particular strains under defined conditions, rather than representing a generalized characteristic of the genus [94].

In contrast, other strains of *Bacillus subtilis* (e.g., strain AS1 and subsp. *inaquosorum*) were primarily reported in the extremophilic dataset as being tolerant to metals such as Cd, Cu, Fe, and Zn under alkaline conditions (pH 8.68; [31]). In these cases, the reported metal concentrations corresponded to exposure levels rather than experimentally demonstrated remediation processes. This distinction is particularly relevant for Bacillus, as its persistence in extreme or metal-rich environments may be partially explained by survival strategies such as sporulation, which do not necessarily imply active metabolic involvement in metal removal.

*Acidithiobacillus ferrooxidans* (formerly *Thiobacillus ferrooxidans*) showed a consistent association with active metal transformation processes, particularly the bioleaching of Fe, Cu, and Cr, under acidic conditions. The reported remediation values included Cr (63 mg/kg), Cu (816.1 mg/kg), and Pb (643.8 mg/kg) [79] under acidic conditions, typically within a pH range of 1.8–2.5 [95,96]. This species is widely recognized for its role in bioleaching processes, typically occurring at pH 1.5–3.5 [97], where Fe- and S-oxidation pathways generate ferric iron and sulfuric acid, enabling both direct and indirect bioleaching, as well as the solubilization of metals associated with sulfide minerals [79,97]. Under controlled conditions, these oxidative processes can contribute to the removal or mobilization of metals as part of engineered treatment systems [27]. However, it is also well-established that *Acidithiobacillus* species play a well-documented role in the generation of AMD in unmanaged environments, accelerating acidification, and metal mobilization. This dual behavior highlights the importance of considering the environmental context when evaluating the remediation potential [27].

Despite its ability to tolerate up to 8000 mg/kg of Fe at pH 2.0, its removal efficiency for some metals remains variable, with values of 4 mg/kg for Cd, 1 mg/kg for Cu, and 65 mg/kg for Zn under specific conditions [70]. This distinction emphasizes that high metal tolerance does not correspond to high remediation efficiency, as effective removal depends on specific metabolic pathways and physicochemical interactions [98]. Furthermore, although *A. ferrooxidans* has been reported to mediate Fe removal (190 mg/kg) at near-neutral pH (7.2) [70], its activity is generally constrained above pH 6.5 [99], which may limit its applicability to acidic conditions.

Overall, these findings indicate that strains of *B. subtilis*, *P. megaterium*, and *A. ferrooxidans* exhibit distinct yet effective mechanisms for metal remediation under a wide range of pH conditions. However, the strength and consistency of the available evidence vary among taxa and are often strain-specific. Therefore, the potential for bioremediation should be interpreted cautiously, considering both the environmental context and the underlying mechanisms involved. These observations suggest that the targeted isolation and characterization of strains adapted to specific conditions may provide more reliable candidates for effective metal remediation in mining-impacted environments [21,35,70,79,99].

### 4.2. Bacillus Subtilis: A Potential Extremophilic Metal-Remediating Bacterium

Among the microorganisms involved in the remediation of mining environments, *B. subtilis* has been widely reported owing to its ecological versatility and frequent occurrence in metal-contaminated systems. Although not considered an obligate extremophile, numerous studies have described extremotolerant strains capable of persisting under a wide range of environmental conditions, including variations in pH, temperature, and metal concentration [21]. In addition, its presence in the rhizosphere and its association with plant growth and stress mitigation have been documented, suggesting a potential role in the management of metal toxicity in contaminated soils [21,100].

Within the dataset analyzed in this review, evidence of active metal remediation by *B. subtilis* was limited and primarily associated with Pb precipitation under specific experimental conditions (Table 4). However, additional studies have reported its involvement in interactions with other metals, including Cd (86.3% reduction; [100], strain KC6), Cu (accumulation of Pb, Cd, and Cu [21], strain 7611), Fe [101] (strain 1427), and Zn [102]. These findings, although not all derived from a systematic dataset, provide complementary evidence supporting the broader metal-interacting capacity of this species and related taxa. One of the primary mechanisms described for *B. subtilis* is biosorption, a process in which functional groups present on bacterial cell surfaces bind to metal ions, facilitating their immobilization and reducing their bioavailability [103]. In addition, some strains have been reported to contribute to metal precipitation by producing extracellular compounds that alter local environmental conditions and promote the formation of insoluble metal complexes [104]. These mechanisms highlight the potential role of B. subtilis in reducing metal mobility in contaminated environments.

Compared to conventional physicochemical remediation approaches, such as thermal treatment, soil washing, and vitrification [90,105,106,107,108,109], microbial-based strategies have been proposed as potentially less disruptive alternatives, as they may enable in situ treatment and reduce the need for extensive soil manipulation. However, the effectiveness of these approaches is highly dependent on environmental conditions, microbial activity, and site-specific factors; therefore, they should be evaluated on a case-by-case basis [110].

The ability of *B. subtilis* strains to persist across a relatively broad pH range (3.0–9.0) and under metal stress conditions further supports their relevance in contaminated environments [111]. Nevertheless, it is important to note that tolerance does not necessarily imply active remediation, and in some cases, survival may be associated with physiological strategies, such as sporulation, rather than continuous metabolic activity [112].

In addition, *Bacillus*-related taxa (including *B. subtilis* and closely related genera such as *Priestia* and *Lysinibacillus*) have been consistently reported across multiple studies in both tolerance and remediation contexts [71,77,81,82,83]. These bacteria are frequently isolated from mining-impacted soils and contaminated environments, where they are exposed to complex mixtures of metals such as Pb, Cd, and Cu [77,82]. Their widespread occurrence and functional versatility highlight their ecological relevance and support their selection as model organisms for bioremediation strategies.

### 4.3. Parameters Influencing Remediation Capacity of Bacteria

As pH is one of the parameters that influence metal content in soil, along with factors such as cation exchange capacity, organic matter, and clay content [113], analyzing the pH during this review was essential. Metal-tolerant bacteria are mostly associated with alkaline environments (pH > 8) [29,31,34]. Alkaline pH increases the formation of metal hydroxides due to the insolubility generated at higher pH levels [114,115], leading to precipitates and reducing metal bioavailability for bacteria [116]. *Deinococcus wulumuqiensis* can tolerate concentrations of 80, 100, and 140 mg/kg (Cd, Cu, and Pb, respectively) in an aqueous medium with an acidic pH of 5.4 [35]; however, its optimal growth pH is between 7.0 and 8.0 [117]. Based on the reported data, the tolerance capacity of this species likely increases with increasing pH.

Additionally, the interaction between pH and metal availability must be considered in the context of multi-metal systems. In mining environments, metals such as Pb, Cd, and Cu often coexist, and their combined behavior can influence both solubility and microbial response, as widely documented in mine waste geochemistry studies [42]. Under alkaline conditions, precipitation processes tend to dominate, particularly for Pb and Cd, due to the formation of insoluble hydroxides and carbonates [37]. In contrast, acidic conditions inherently favor bioleaching mechanisms, especially in systems influenced by AMD, where iron- and sulfur-oxidizing microorganisms—such as *Acidithiobacillus ferrooxidans* and *Acidithiobacillus thiooxidans*—actively enhance metal solubilization [30,31,43,97].

Our dataset supports this bimodal environmental behavior. While some bacterial taxa show increased metal exposure capacities with increasing pH [30,45,74], bioleaching-based metal recovery decreases significantly with increasing pH [69,70,71]. This physiological efficiency under extreme acidity is well exemplified by *A. ferrooxidans*, which can tolerate up to 8000 mg/kg of Fe at pH 2 while maintaining high bioleaching yields [30], operating optimally within a narrow acidic range (e.g., pH 0.9) compared to neutral conditions [70,73,79]. This highlights that bacterial remediation is not only metal-specific but also strictly dependent on the geochemical context, which ultimately regulates both microbial metabolic activity and metal mobility [42].

Recent studies have reinforced the central role of bacteria in the remediation of metal-contaminated environments, supporting the trends observed in this review. Research on mining effluents and agricultural soils continues to demonstrate that indigenous bacterial communities and newly isolated strains can withstand variable multi-metal pressure and have high bioremediation potential [118,119,120]. Functional genomic analyses have further clarified these molecular mechanisms, such as the chromate-remediation pathways identified in *Bacillus cereus* BC4 [121]. Furthermore, recent evidence highlights that metal-transforming bacterial consortia can substantially reduce Cd and Pb bioavailability and plant uptake [122], whereas beneficial inoculants enhance phytoremediation efficiency by modifying rhizosphere structure and metal mobility [123,124]. Collectively, this body of evidence strengthens the conclusion that metal-resistant bacteria play a decisive role in driving biogeochemical metal transformations and improving remediation outcomes across diverse contaminated matrices.

### 4.4. Research Prospects

The ability of microorganisms to associate with the rhizosphere of plants and enhance metal accumulation has been previously reported [76,80]. In particular, strains of *Pseudomonas putida* associated with *Trifolium repens* increased the total absorption of metals, such as Cd, Cr, and Pb, by 575% [76]. Similarly, strains of *Serratia marcescens* have been reported to be associated with *Robinia pseudoacacia* (L.) and *Sophora xanthantha*, improving the absorption of up to 22.3 mg/L of Cd and 19.5 mg/L of Pb in the aerial parts [80]. These findings highlight the importance of plant–microbe interactions in enhancing phytoremediation processes. Investigating the specificity of these associations between bacterial strains and plant species is crucial for developing integrated remediation strategies for metal-contaminated environments [125]. Further research on the role of *B. subtilis* in contaminated soils should focus on identifying and characterizing strains with demonstrated remediation capacity, as well as on optimizing environmental conditions that enhance their activity. Expanding this line of research may contribute to the development of more targeted and effective strategies for the rehabilitation of mining-impacted ecosystems.

### 4.5. Limitations of the Study

Although this systematic review provides a rigorous synthesis of the evidence regarding metal-interacting bacteria in mining environments, several methodological limitations must be acknowledged. First, regarding the search architecture, our strategy was strictly tailored to capture precise microbiological taxa and specific mining-impacted geochemical settings. As noted during the evaluation process, this high specificity may have inadvertently excluded broader ecological studies or grey literature, where bacterial tolerance mechanisms are mentioned only peripherally [48]. Furthermore, restricting to peer-reviewed literature and the lack of access to a few full-text documents may have led to the omission of potentially relevant unpublished or industry-driven reports.

Additionally, the systematic protocol was not prospectively registered in public registries (e.g., OSF or PROSPERO) prior to the formal data extraction phase, which represents a limitation in terms of pre-registered transparency, although strict adherence to the PRISMA guidelines was maintained throughout the execution of this review.

Second, a quantitative meta-analysis of the intervention outcomes was deemed unfeasible because of the severe methodological heterogeneity and low-quality profiles identified across the primary literature during our formal risk of bias assessment (Tables S1 and S2). The included studies exhibited a profound lack of standardization in critical experimental parameters, such as the frequent omission of parallel sterile abiotic controls in removal assays––which precludes the mathematical isolation of microbially mediated mechanisms from spontaneous chemical precipitation–– as well as highly variable metal exposure metrics (nominal vs. instrumentally verified dosing) and a widespread absence of independent biological replication (*n* < 3).

Consequently, attempting quantitative statistical aggregation under these conditions would introduce significant bias and lead to misleading effect-size estimations. Therefore, our synthesis was restricted to a qualitative and semi-quantitative framework, highlighting that the current body of literature in environmental microbiology requires a critical shift toward elevated methodological standardization before robust quantitative global consolidations can be achieved.

## 5. Conclusions

Studies on metal-tolerant and metal-interacting bacteria in mining environments highlight their potential roles in the bioremediation of contaminated systems. However, this systematic review reveals a significant knowledge gap: the evidence base bridging pure metal tolerance and demonstrated remediation capacity is remarkably sparse.

When cross-referencing our systematic datasets, only two species (*Bacillus subtilis* and *Acidithiobacillus ferrooxidans*) appeared in both tolerance and active remediation studies. Rather than identifying these as universal representative species, this remarkably low overlap constitutes a critical and negative finding. This demonstrates that while diverse bacterial taxa exhibit survival strategies under extreme metal stress, most metal-tolerant extremophiles lack a rigorously demonstrated remediation capacity. The current literature frequently conflates mere persistence in metal-rich environments with functional remediation, highlighting a severe disconnect in environmental microbiology research.

Furthermore, our risk of bias assessment underscores that this knowledge gap is exacerbated by methodological shortcomings across the literature. The widespread heterogeneity in experimental designs––particularly the frequent omission of sterile abiotic controls in removal assays––limits the ability to differentiate true microbially mediated remediation from spontaneous chemical precipitation. As this review highlights, parameters such as pH profoundly regulate both metal availability and microbial activity, meaning that uncontrolled confounding factors severely weaken the current evidence base.

Consequently, there is an urgent need for more standardized experimental validation under environmentally relevant conditions. Future research must shift from merely isolating and cataloging metal-tolerant strains to rigorously testing their active remediation efficiencies using standardized baseline controls, adequate replicates, and reliable analytical measurements. Ultimately, advancing microbial and plant-based bioremediation strategies in mining-impacted ecosystems will depend not on finding universally tolerant species, but on elevating the methodological rigor required to prove their functional applicability.

## Figures and Tables

**Table 1:** Inclusion and exclusion criteria for the review.

Criteria	Extremophilic Microorganisms	Remediation Bacteria
Inclusion	Studies that specified metal concentrations and identified tolerant extremophilic or extremotolerant bacteria.	Studies that identified bacteria involved in metal remediation
Exclusion	Studies focused on microorganisms that are neither extremophilic nor extremotolerant, or environments not characterized by high metal content	Studies not centered on microorganisms or lacking a remediation focus
		Studies focused on non-mining environments

**Table 2:** Articles included in the qualitative synthesis of extremophilic or extremotolerant bacteria in environments with high metal content after applying the inclusion and exclusion criteria.

N	Author(s)	Year	Title	Metals Reports	DOI or PMID
1	Matlakowska et al. [30]	2006	The growth, ferrous iron oxidation, and ultrastructure of *Acidithiobacillus ferrooxidans* in the presence of dibutyl phthalate	Fe	PMID: 17338273
2	Wu et al. [57]	2007	Isolation and identification of metal-resistant iron-oxidizing bacteria	Cu and Pb	10.1007/BF03403359
3	Moreno et al. [31]	2012	Analysis and characterization of cultivable extremophilic hydrolytic bacterial community in heavy metal-contaminated soils from the Atacama Desert and their biotechnological potentials	Cd, Cu, Fe and Zn	10.1111/j.1365-2672.2012.05366.x
4	Tomova et al. [58]	2014	Characterization of heavy metals resistant heterotrophic bacteria from soils in the Windmill Islands region, Wilkes Land, East Antarctica	Cr, Cu, Pb and Zn	10.2478/popore-2014-0028
5	Bafana et al. [59]	2015	Mercuric reductase activity of multiple heavy metal-resistant *Lysinibacillus sphaericus* G1	Cd, Cr, Zn	10.1002/jobm.201300308
6	Ramanathan y Ting [34]	2016	Alkaline bioleaching of municipal solid waste incineration fly ash by autochthonous extremophiles	Cd, Cr, Cu, Fe, Pb and Zn	10.1016/j.chemosphere.2016.06.055
7	Asatiani et al. [60]	2018	Effect of the simultaneous action of zinc and chromium on *Arthrobacter* spp.	Cr and Zn	10.1007/s11270-018-4046-0
8	Abbaszade et al. [61]	2020	Whole genome sequence analysis of *Cupriavidus campinensis* S14E4C, a heavy metal resistant bacterium	Pb	10.1007/s11033-020-05490-8
9	Gallo et al. [33]	2021	Bioprospecting of extremophilic microorganisms to address environmental pollution	Cd, Cr and Cu	10.3791/63453
10	Sher et al. [62]	2021	Characterization of multiple metal resistant Bacillus licheniformis and its potential use in arsenic contaminated industrial wastewater	Cd, Cr, Cu, Pb and Zn	10.1007/s13201-021-01407-3
11	Dai et al. [28]	2021	Colonized extremophile * Deinococcus radiodurans* alleviates toxicity of Cd and Pb by suppressing heavy metal accumulation and improving antioxidant system in rice	Cd and Pb	10.1016/j.envpol.2021.117127
12	Diba et al. [29]	2021	Isolation and characterization of halophilic bacteria with the ability of heavy metal bioremediation and nanoparticle synthesis from the Khara Salt Lake in Iran	Pb	10.1007/s00203-021-02380-w
13	Xie et al. [35]	2021	Removal of Cu and Pb ions from water using the extremophile *Deinococcus wulumuqiensis* R12	Cr, Cu and Pb	10.5004/dwt.2021.27338
14	Ausuri et al. [63]	2022	Bioremediation of multiple heavy metals mediated by Antarctic marine isolate Dietzia psychralcaliphila JI1D	Cd, Cr, Cu, Pb and Zn	10.3390/jmse10111669
15	Patel et al. [64]	2022	Cadmium-tolerant plant growth-promoting bacteria *Curtobacterium oceanosedimentum* improves growth attributes and strengthens antioxidant system in chili (*Capsicum frutescens*)	Cd	10.3390/su14074335
16	Yang et al. [65]	2023	Effect of anthropogenic disturbances on the microbial relationship during bioremediation of heavy metal-contaminated sediment	Cd, Cu and Zn	10.3390/microorganisms11051185
17	Rosas-Ramírez et al. [66]	2023	Identification of halophilic bacteria tolerant to heavy metals	Cr and Pb	10.20937/RICA.54220
18	Qiu et al. [32]	2023	Molecular insights into a novel Cu(I)-sensitive ArsR/SmtB family repressor in extremophile *Acidithiobacillus caldus*	Cu	10.1128/aem.01266-22
19	Wang et al. [67]	2024	Surface display of multiple metal-binding domains in *Deinococcus radiodurans* alleviates cadmium and lead toxicity in rice	Cd and Pb	10.3390/ijms252312570
20	Panyushkina et al. [68]	2025	Mechanisms of microbial hyper-resistance to heavy metals: Cellular metal accumulation, metabolic reorganization, and GroEL chaperonin in extremophilic bacterium *Sulfobacillus thermotolerans* in response to zinc	Cu, Pb and Zn	10.1016/j.jhazmat.2025.137490

**Table 3:** Articles included in the qualitative synthesis after applying inclusion and exclusion criteria in the search for metal-remediating bacteria in mining-related environments.

N	Author(s)	Year	Title	Metals Reports	DOI or PMID
1	Groudev et al. [69]	2001	Bioremediation of a soil contaminated with radioactive elements	Cu and Pb	10.1016/S0304-386X(00)00187-0
2	Hulshof et al. [70]	2003	Microbial and nutrient investigations into the use of in situ layers for treatment of tailings effluent	Cu, Fe and Zn	10.1021/es020822r
3	Pruden et al. [71]	2007	The effect of inoculum on the performance of sulfate-reducing columns treating heavy metal contaminated water	Cd and Zn	10.1016/j.watres.2006.11.025
4	Kang et al. [72]	2015	Bioremediation of lead by ureolytic bacteria isolated from soil at abandoned metal mines in South Korea	Pb	10.1016/j.ecoleng.2014.10.009
5	Nicolova et al. [73]	2017	Microbial removal of toxic metals from a heavily polluted soil	Cd, Cu and Zn	10.1016/j.gexplo.2016.11.003
6	Chang et al. [74]	2019	Cr(VI) removal performance from aqueous solution by *Pseudomonas* sp. strain DC-B3 isolated from mine soil: characterization of both Cr(VI) bioreduction and total Cr biosorption processes	Cr	10.1007/s11356-019-06017-w
7	Zhu et al. [75]	2019	The immobilization effects on Pb, Cd and Cu by the inoculation of organic phosphorus-degrading bacteria (OPDB)with rapeseed dregs in acidic soil	Cd, Cu and Pb	10.1016/j.geoderma.2019.04.015
8	Liu et al. [76]	2021	Endophyte *Pseudomonas putida* enhanced Trifolium repens L. growth and heavy metal uptake: A promising in situ non-soil cover phytoremediation method of nonferrous metallic tailing	Cd, Cr, and Pb	10.1016/j.chemosphere.2021.129816
9	Oyetibo et al. [77]	2021	Microbiome of highly polluted coal mine drainage from Onyeama, Nigeria, and its potential for sequestrating toxic heavy metals	Cd and Pb	10.1038/s41598-021-96899-z
10	Liu et al. [78]	2022	Experimental study on treatment of heavy metal-contaminated soil by manganese-oxidizing bacteria	Cd, Cu, Pb, and Zn	10.1007/s11356-021-15475-0
11	Sur et al. [79]	2022	Extraction of metals from polluted soils by bioleaching in relation to environmental risk assessment	Cr, Cu and Pb	10.3390/ma15113973
12	Zheng et al. [80]	2023	Enhancing remediation potential of heavy metal contaminated soils through synergistic application of microbial inoculants and legumes	Cd and Pb	10.3389/fmicb.2023.1272591
13	Han et al. [81]	2023	Stabilization of Pb(II) in wastewater and tailings by commercial bacteria through microbially induced phosphate precipitation (MIPP)	Pb	10.1016/j.scitotenv.2023.161628
14	Hu et al. [82]	2024	Genomic characterization of a novel ureolytic bacteria, *Lysinibacillus capsici* TSBLM, and its application to the remediation of acidic heavy metal-contaminated soil	Cu and Pb	10.1016/j.scitotenv.2024.172170
15	Ghosh et al. [83]	2025	Augmented elimination of cadmium and mercury by *Cytobacillus firmus* and *Paenibacillus massiliensis* isolated from heavy metal contaminated soil samples	Cd	10.1016/j.bcab.2025.103669
16	Yang et al. [84]	2025	Effect of urea concentration on the combined pollution of Cd and Ni in microbiologically induced calcite precipitation (MICP) treatment	Cd	10.1007/s10532-025-10204-7

**Table 4:** Summary of operational pH ranges, metal interaction mechanisms, and active remediation concentrations (mg·kg^−1^) reported for heavy metal-remediating bacteria across the analyzed literature.

N	Family	Genus	Specie ‡	Environment	Metal Interaction	pH	Cd	Cr	Cu	Fe	Pb	Zn	Ref.
1	Acidithiobacillaceae	*Acidithiobacillus*	*A. ferrooxidans*	Acid drainage	Bioleaching	7.2	4	-	1	190	-	65	[70]
2	Acidithiobacillaceae	*Acidithiobacillus*	*A. ferrooxidans* ^a^	Mining soils/Cinnamon forest soils	Precipitation/Bioleaching	2.80–5.35	-	63	140–816.11	-	643.8	168	[79,73]
3	Acidithiobacillaceae	*Acidithiobacillus*	*A. thiooxidans*	Acid drainage	Bioleaching	7.2	4	-	1	190	-	65	[70]
4	Bacillaceae	*Bacillus*	*B. cereus*	Mine drainages	Precipitation	8.2	139.3	-	-	-	593.3	-	[77]
5	Bacillaceae	*Bacillus*	*B. subtilis*	Mining tailings	Precipitation	3.00–9.00	-	-	-	-	250–495.0	-	[81]
6	Bacillaceae	*Cytobacillus*	*Cytobacillus firmus* (strain BS4)	Industrial soils	Biosorption	7	19	-	-	-	-	-	[83]
7	Bacillaceae	*Lysinibacillus*	*Lysinibacillus capsici* (strain TSBLM)	Mining soils	Precipitation	5.16	-	-	18	-	28.8	-	[82]
8	Bacillaceae	*Priestia*	*P. megaterium* ^b^	Mining tailings	Precipitation	3.00–9.00	-	-	-	-	250–420.5	-	[81]
9	Desulfitobacteriaceae	*Desulfosporosinus*	*Desulfosporosinus acidianus*	Cinnamon forest soils	Precipitation/Bioleaching	3	4.4	-	-	-	-	-	[73]
10	Desulfobacteriaceae	*Desulfobacterium*	*-*	Acid drainage	Bioleaching	6	0.24	-	-	-	-	0.43	[71]
11	Enterobacteriaceae	*-*	*-*	Mine drainages	Precipitation	8.2	139.3	-	-	-	593.3	-	[77]
12	Enterobacteriaceae	*Enterobacter cloacae strain KJ-46*		Mining soils	Precipitation	7	-	-	-	-	3.2–4.9 mg/L	-	[72]
13	Enterobacteriaceae	*Enterobacter* sp. *strains SX4*	*Enterobacter* sp. (strain SX4)	Cinnamon agricultural soils	Precipitation	7	0.9	-	-	-	-	-	[84]
14	Enterobacteriaceae	*Serratia*	*S. marcescens*	Mining tailings	Immobilization	5.60–9.96	0.04–35	-	6.0–40.0	-	4.0–156	-	[75,80]
15	Exiguobacteriaceae	*Exiguobacterium*	*E. aurantiacum*	Mine drainages	Precipitation	8.2	139.3	-	-	-	593.3	-	[77]
16	Halothiobacillaceae	*Halothiobacillus*	*H. neapolitanus* ^c^	Agricultural soils *	Immobilization	7.59	2.4	-	260	-	123	-	[69]
17	Hydrogenophilaceae	*Thiobacillus*	*T. thioparus*	Acid drainage	Bioleaching/Immobilization	7.2	2.3–4.0	-	1.0–260	190	123	65	[70,69]
18	Hydrogenophilaceae	*Thiobacillus*	*T. denitrificans*	Agricultural soils *	Immobilization	7.59	2.4	-	260	-	123	-	[69]
19	Leptospirillaceae	*Leptospirillum*	*L. ferrooxidans*	Cinnamon forest soils	Precipitation/Bioleaching	3	-	-	140	-	-	-	[73]
20	Moraxellaceae	*Acinetobacter*	*A. pittii*	Mine drainages	Precipitation	8.2	139.3	-	-	-	593.3	-	[77]
21	Nocardiopsaceae	*Nocardiopsis*	*Nocardiopsis* sp. (strain TXV7-8SG2)	Lake Texcoco, Mexico	Exposed†	7	-	65,000	-	-	1600	-	[71]
22	Paenibacillaceae	*Paenibacillus*	*P. massiliensis* (strain BS10)	Industrial soils	Biosorption	7	16	-	-	-	-	-	[83]
23	Planococcaceae	*Sporosarcina*	*S. koreensis*	Mine drainages	Precipitation	8.2	139.3	-	-	-	593.3	-	[77]
24	Pseudomonaceae	*Pseudomonas*	*P. taiwanensis* (strain ZM11)	Agricultural soils *	Precipitation/Biosorption	6.5–7.0	12.5–20	-	30–45	-	45–75	16–28	[78]
25	Pseudomonadaceae	*Pseudomonas*	*P. citronellolis*	Mine drainages	Precipitation	8.2	139.3	-	-	-	593.3	-	[77]
26	Pseudomonadaceae	*Pseudomonas*	*P. putida*	Mining tailings	Biosorption	7.6	3	9	-	-	10	-	[76]
27	Pseudomonadaceae	*Pseudomonas*	*Pseudomonas* sp.	Mining soils	Biosorption	2	-	32	-	-	-	-	[74]
28	Xanthobacteraceae	*Starkeya*	*S. novella* ^d^	Agricultural soils *	Immobilization	7.59	2.3	-	260	-	123	-	[69]

* Agricultural soils near mining areas. ‡ Taxonomic names updated according to current nomenclature. The following species were reported under different names in the original publications: *Thiobacillus ferrooxidans* (^a^) [85], *Bacillus meaterium* (^b^) [86,87], *Thiobacillus neapolitanus* (^c^) [85] and *Thiobacillus novellus* (^d^) [88]. (a)–(d) Names as originally reported in the cited studies.

## Data Availability

The original contributions presented in this study are included in the article/Supplementary Material. Further inquiries can be directed to the corresponding author.

## Supplementary Materials

The following supporting information can be downloaded at: https://www.mdpi.com/article/10.3390/microorganisms14061312/s1. Table S1. Methodological quality and risk of bias assessment criteria for studies investigating heavy metal tolerance mechanisms (*n* = 20). Table S2. Methodological quality and risk of bias assessment criteria for studies investigating bacterial bioremediation and heavy metal removal efficiency (*n* = 16). Table S3. Metal-tolerant bacteria identified in the analyzed studies, including environmental or experimental exposure to metal concentrations (mg·kg^−1^) and associated pH conditions. Table S4. Metal-remediating bacteria reported in the analyzed studies, including experimentally measured remediation concentrations (mg·kg^−1^) and the pH conditions under which remediation occurred. 
