# Supplementary material for: Metals and Extremophilic Bacteria in Mining Environments: A Systematic Review
Source: Microorganisms. 2026 Jun 11;14(6):1312. doi: 10.3390/microorganisms14061312 (PMC13303428; doi:10.3390/microorganisms14061312)
Supplement: Supplementary file 1 [file microorganisms-14-01312-s001.zip › microorganisms-4221334-supplementary.pdf]

Supplementary Data

Table S1. Methodological quality and risk of bias assessment criteria for studies investigating heavy metal tolerance mechanisms (n = 20).

N	Author(s)	Year	D1	D2	D3	D4	D5	Overall	Main Justification
1	Matlakowska *et al*.	2006	+	+	+	+	+	+	Low risk across all domains.
2	Wu *et al*.	2007	+	?	+	+	-	-	D2 and D5: Metal concentrations were calculated nominally (lacking instrumental verification). Most critically, independent biological replicates and statistical analyses were entirely omitted from the kinetic growth curves.
3	Moreno *et al*.	2012	+	+	+	+	+	+	Low risk across all domains.
4	Tomova *et al*.	2014	+	?	+	+	-	-	D2 and D5: The minimum inhibitory concentration (MIC) was determined theoretically on agar plates without verifying potential metal precipitation, and experimental replicates were entirely omitted from the methodology.
5	Bafana *et al*.	2015	+	?	+	+	+	+	D2 (?): Initial metal dosing was determined theoretically; however, the overall low-risk profile was maintained due to robust sterile abiotic controls, instrumental analytical measurement of residual metals, and sound statistical replication (n = 3).
6	Ramanathan y Ting	2016	+	+	?	+	+	+	D3 (?): The use of an autochthonous/native mixed culture isolated directly from fly ash limits universal genetic traceability, restricting the ability of external laboratories to precisely replicate the experiment with the exact same microbial composition.
7	Asatiani *et al*.	2018	+	+	+	+	+	+	Low risk across all domains.
8	Abbaszade *et al*.	2020	+	?	+	+	+	+	D2 (?): Nominal dosing. Since this study primarily focused on whole-genome sequencing (WGS), the phenotypic metal tolerance assays relied on theoretical concentrations without using analytical instrumentation (e.g., AAS/ICP) to verify the actual initial exposure. The overall low-risk rating is preserved due to complete genetic traceability (D3) and robust statistical replication.
9	Gallo *et al*.	2021	+	+	+	+	+	+	Low risk across all domains.
10	Sher *et al*.	2021	+	-	+	+	+	-	D2: Arsenic exposure and removal were quantified using indirect colorimetry (molybdenum blue method) rather than a more robust, standard analytical approach such as ICP or AAS.
11	Dai *et al*.	2021	+	+	+	+	+	+	Low risk across all domains.
12	Diba *et al*.	2021	+	+	+	+	+	+	Low risk across all domains.
13	Xie *et al*.	2021	+	+	+	+	+	+	Low risk across all domains.
14	Asuri *et al*.	2022	+	+	+	+	+	+	Low risk across all domains.
15	Patel *et al*.	2022	+	?	+	+	+	+	D2 (?): Theoretical nominal dosing. The actual initial metal concentration in the assay plates was not analytically or instrumentally verified.
16	Yang *et al*.	2023	-	+	+	+	+	-	D1: The bioleaching experimental design lacked a sterile abiotic control (sterile sediment without bacterial inoculation), precluding differentiation between microbially mediated processes and purely abiotic chemical interactions.
17	Rosas-Ramírez *et al*.	2023	+	?	+	+	?	?	D2 and D5: Characterized by a lack of analytical quantification of initial metal exposure (D2) and an absence of independent biological replicates in the treatment curves (n = 1).
18	Qiu *et al*.	2023	+	?	+	+	+	+	D2 (?): Moderate risk in initial exposure measurement due to nominal calculations; however, this is compensated for by absolute rigor in genetic sequencing validation and statistical analyses.

N	Author(s)	Year	D1	D2	D3	D4	D5	Overall	Main Justification
19	Wang *et al*.	2024	+	+	+	+	+	+	Low risk across all domains.
20	Panyushkina *et al*.	2025	+	+	+	+	+	+	Low risk across all domains.
Risk %		Low	95	60	95	100	85	75	
		Moderate	0	35	5	0	5	5	
		High	5	5	0	0	10	20	

Table S2. Methodological quality and risk of bias assessment criteria for studies investigating bacterial bioremediation and heavy metal removal efficiency (n = 16).

N	Author(s)	Year	D1	D2	D3	D4	D5	Overall	Main Justification
1	Groudev *et al*.	2001	-	?	?	?	-	-	D1, D2, and D5: This large-scale lysimeter assay lacked sterile abiotic controls (D1), precluding the isolation of active bioleaching from physical water-driven leaching. Additionally, the standardization of the initial inoculum was omitted (D2), and metal reduction rates were reported as single absolute values lacking independent replicates or standard deviation (D5).
2	Hulshof *et al*.	2003	-	?	+	+	-	-	D1 and D5: Complete absence of sterile abiotic control columns, making it impossible to rule out metal reduction via physical adsorption onto the wood matrix rather than biological mechanisms (D1). Furthermore, the experiment was conducted without replication (n = 1 per treatment), invalidating statistical significance of the reported reduction rates (D5).
3	Pruden *et al*.	2007	+	?	+	+	?	?	D2 and D5: Although the study featured robust analytical validation (ICP-AES) and an appropriate abiotic control design (D1), it failed to standardize or report the initial cell density of the complex inoculum (D2). Moreover, assays were performed only in duplicate (n = 2), failing to meet the minimum statistical threshold of three independent replicates to ensure data consistency and dispersion analysis (D5).
4	Kang *et al*.	2015	+	?	+	?	+	?	D2 and D4: A study with robust analytical performance (ICP-OES) and controlled design (D1), but presenting procedural omissions: it failed to standardize the initial cell density (OD600) of the inoculum in the primary biomineralization assay (D2) and did not monitor or report pH fluctuations, which represent a critical confounding variable in lead precipitation mechanisms (D4).
5	Nicolova *et al*.	2017	-	?	+	+	-	-	D1, D2, and D5: Similar to other large-scale greenhouse experiments, no sterile abiotic control plots were established (D1), and the bacterial inoculum load was not standardized (D2). Scale constraints also resulted in a total lack of statistical replication (n≥3), reporting single metal extraction outcomes without variance validation (D5).
6	Chang *et al*.	2019	+	+	+	+	+	+	Low risk across all domains.
7	Zhu *et al*.	2019	+	+	+	+	+	+	Low risk across all domains.
8	Liu *et al*.	2021	+	+	+	+	+	+	Low risk across all domains.
9	Oyetibo *et al*.	2021	-	-	+	+	+	-	D1 and D2: Critical experimental design flaws consisting of a complete lack of an uninoculated abiotic control group, preventing the mathematical differentiation of urease-mediated biomineralization from spontaneous chemical precipitation (D1). Additionally, it omits the standardization of the initial inoculated biomass density (D2).
10	Liu *et al*.	2022	+	+	+	+	+	+	Low risk across all domains.
11	Sur *et al*.	2022	-	?	+	+	-	-	D1, D2, and D5: This soil bioleaching study did not utilize sterile abiotic controls, making it impossible to separate active

N	Author(s)	Year	D1	D2	D3	D4	D5	Overall	Main Justification
									biological extraction from natural chemical dissolution caused by the extreme acidity of the medium (D1). It also fails to quantify the applied biomass (D2), and the removal efficiencies are reported without independent replicates or standard error bars (D5).
12	Zheng *et al.*	2023	+	?	+	+	+	+	Low risk across all domains.
13	Han *et al.*	2023	+	+	+	+	+	+	Low risk across all domains.
14	Hu *et al.*	2024	+	+	+	+	+	+	Low risk across all domains.
15	Ghosh *et al.*	2025	-	+	+	+	+	-	D1: Despite correct analytical and statistical replication, a severe procedural omission occurred by not including an abiotic control flask (medium containing the metal without bacterial inoculation). Without this blank, it is impossible to verify whether metal removal was strictly biological or driven by volatilization and wall adherence inside the flask.
16	Yang *et al.*	2025	+	+	+	+	?	+	D5 (?): Moderate risk due to ambiguous reporting details on statistical dispersion at certain kinetic points, although the study maintained an overall robust methodological quality profile.
Risk %		+	62,5	50	93,75	87,5	62,5	50	
		?	0	43,75	6,25	12,5	12,5	12,5	
		-	37,5	6,25	0	0	25	37,5	

Table S3. Metal-tolerant bacteria identified in the analyzed studies, including environmental or experimental exposure to metal concentrations (mg·kg⁻¹) and associated pH conditions.

N	Author/s	Family	*Species*	pH	Cd	Cr	Cu	Fe	Pb	Zn
1	Ramanathan and Ting (2016)	Erythrobacteraceae	*P. donghaensis* strain TRTYP1	10.01	610	130	31.85	8960	1420	3290
2		Carnobacteriaceae	*Alkalibacterium sp.* strain TRTYP6	10.01	610	130	31.85	8960	1420	3290
3		Cyclobacteriaceae	*Fontibacter sp.* strain TRTYP16	10.01	610	130	31.85	8960	1420	3290
4		Microbacteriaceae	*M. alkaliphila* strain TRTYP4	10.55	610	1930	530	48.56	1390	2360
5		Carnobacteriaceae	*A. pelagium* strain TRTYP5	10.55	610	1930	530	48.56	1390	2360
6		Caryophanaceae	*B. cecembensis* strain TRTYP8	10.55	610	1930	530	48.56	1390	2360
7		Carnobacteriaceae	*A. indicireducens* strain TRTYP18	10.55	610	1930	530	48.56	1390	2360
8		Bacillaceae	*B. anthracis* strain TRTYP13	11.98	610	1920	530	42.29	1390	2400
9		Bacillaceae	*B. firmus* strain TRTYP7	8.42	600	1690	400	21.97	1360	2070
10		Bacillaceae	*B. pumilus* strain TRTYP2	8.74	600	1690	400	21.97	1360	2070
11		Microbacteriaceae	*A. aurantiacus* strain TRTYP3	8.74	600	1690	400	21.97	1360	2070
12		Bacillaceae	*B. horikoshii* strain TRTYP10	8.74	600	1690	400	21.97	1360	2070
13		Bacillaceae	*B. vietnamensis* strain TRTYP15	8.74	600	1690	400	21.97	1360	2070

N	Author/s	Family	*Species*	pH	Cd	Cr	Cu	Fe	Pb	Zn
14		Bacillaceae	*B. aquimaris* strain TRTYP9	8.94	600	1870	340	-	1320	1900
15		Bacillaceae	*B. licheniformis* strain TRTYP11	8.94	600	1870	340	-	1320	1900
16		Bacillaceae	*B. marisflavi* strain TRTYP12	8.94	600	1870	340	-	1320	1900
17		Bacillaceae	*B. foraminis* strain TRTYP17	8.94	600	1870	340	-	1320	1900
18		Bacillaceae	*B. infantis* strain TRTYP14	9.42	600	1890	430	31.78	1260	2200
19	Moreno *et al*. (2021)	Bacillaceae	*B. seohaeanensis*	8.68	56.21	-	31.78	111.70	-	261.52
20		Bacillaceae	*B. seohaeanensis* strain DV2	8.68	56.21	-	254.20	223.40	-	65.38
21		Bacillaceae	*B. seohaeanensis* strain DV3	8.68	56.21	-	63.55	111.70	-	196.14
22		Bacillaceae	*B. subtilis subsp. inaquosorum*	8.68	337.23	-	254.20	223.40	-	196.14
23		Bacillaceae	*B. subtilis* strain AS1	8.68	56.21	-	127.10	111.70	-	32.69
24		Bacillaceae	*B. stratosphericus*	8.68	337.23	-	254.20	223.40	-	196.14
25		Bacillaceae	*B. oceanisediminis*	8.68	56.21	-	63.55	223.40	-	261.52
26		Bacillaceae	*B. mojavensis*	8.68	56.21	-	63.55	111.70	-	32.69
27		Bacillaceae	*B. licheniformis*	8.68	56.21	-	63.55	167.55	-	32.69
28		Pseudomonadaceae	*P. halophila*	8.68	56.21	-	31.78	27.93	-	32.69
29		Bacillaceae	*H. hunanensis*	8.68	56.21	-	31.78	27.93	-	32.69
30		Bacillaceae	*H. hunanensis* strain AS5	8.68	56.21	-	31.78	27.93	-	32.69
31		Bacillaceae	*H. profundi*	8.68	56.21	-	31.78	27.93	-	32.69
32		Bacillaceae	*H. profundi* strain AS7	8.68	56.21	-	254.20	27.93	-	32.69
33		Bacillaceae	*H. profundi* strain AS8	8.68	56.21	-	31.78	223.40	-	32.69
34		Bacillaceae	*H. profundi* strain AS9	8.68	56.21	-	254.20	27.93	-	32.69
35		Bacillaceae	*H. profundi* strain AS10	8.68	56.21	-	31.78	55.85	-	32.69
36		Bacillaceae	*T. devorans*	8.68	56.21	-	190.65	223.40	-	32.69
37		Bacillaceae	*T. devorans* strain AS12	8.68	337.23	-	31.78	223.40	-	32.69
38		Bacillaceae	*T. devorans* strain AS13	8.68	56.21	-	254.20	27.93	-	32.69
39		Bacillaceae	*T. devorans* strain AS14	8.68	44.64	-	190.65	223.40	-	32.69
40		Bacillaceae	*T. devorans* strain S15	8.68	56.21	-	254.20	27.93	-	130.76
41		Halomonadaceae	*H. organivorans*	8.68	56.21	-	127.10	223.40	-	32.69
42		Staphylococcaceae	*S. roseus*	8.68	56.21	-	127.10	27.93	-	65.38
43		Staphylococcaceae	*S. roseus* strain AS18	8.68	56.21	-	127.10	27.93	-	32.69
44	Dai *et al*. (2021)	Deinococcaceae	*D. radiodurans* strain Δdr2577	7.50	0.01	-	-	-	0.05	-

N	Author/s	Family	*Species*	pH	Cd	Cr	Cu	Fe	Pb	Zn
45	Matlakowska *et al*. (2006)	Acidithiobacillaceae	*A. ferrooxidans*	2.00	-	-	-	8000	-	-
46	Gallo *et al*. (2021)	Bacillaceae	*G. astearothermophilus*	5.50	101.17	13.00	260.56	-	-	-
47		Alicyclobacillaceae	*A. mali*	5.50	89.28	-	31.78	-	-	-
48	Diba *et al*. (2021)	Bacillaceae	*Bacillus sp.* strain A21	7.40	-	-	-	-	1497.6	-
49		Bacillaceae	*Oceanobacillus sp.* strain A22	7.40	-	-	-	-	850.9	-
50		Staphylococcaceae	*Salinicoccus sp.* strain A43	7.40	-	-	-	-	1388.4	-
51	Qiu *et al*. (2023)	Acidithiobacillaceae	*A. caldus*	7.40	-	-	10	-	-	-
52	Xie *et al*. (2023)	Deinococcaceae	*D. wulumuqiensis* strain R12	5.40	-	80.00	100	-	140	-
53	Wang *et al*. (2024)	Deinococcaceae	*Deinococcus radiodurans*	-	112	-	-	-	207	-
54	Panyushkina *et al*. (2025)	Sulfobacillaceae	*Sulfobacillus thermotolerans*	1.8	-	-	5100	-	414	65500
55	Ausuri *et al*. (2022)	Dietziaceae	*Dietzia psychralcaliphila* strain JI1D	9	224	26	64	-	310	65
56	Patel *et al*. (2022)	Microbacteriaceae	*Curtobacterium oceanosedimentum*	-	300	-	-	-	-	-
57	Tomova *et al*. (2014)	Bacillaceae	*Bacillus pumilus* strain Vi-1	7	-	-	-	-	3270	-
58		Bacillaceae	*Bacillus safensis* strain Vi-2	7	-	-	-	-	-	680
59		Bacillaceae	*Bacillus amyloliquefaciens* strain Vi-3	7	-	-	510	-	-	-
60		Bacillaceae	*Lysinibacillus fusiformis* strain Vi-7	7	-	176	-	-	-	-
61	Sher *et al*. (2021)	Bacillaceae	*Bacillus licheniformis*	7--8	200	300	250	-	500	350
62	Yang *et al*. (2023)	Acidithiobacillaceae	*Acidithiobacillus ferrooxidans*	0.9	550	-	860	-	-	960
63		Acidithiobacillaceae	*Acidithiobacillus thiooxidans*	0.9	450	-	780	-	-	940
64	Asatiani *et al*. (2018)	Micrococcaceae	*Pseudarthrobacter oxydans (Arthrobacter oxydans)*	7	-	100	-	-	-	50
65		Micrococcaceae	*Arthrobacter globiformis*	7	-	100	-	-	-	50
66	Rosas-Ramírez *et al*. (2023)	Halomonadaceae	*Halomonas sp.* strain TXO4B-1SG9	8	-	-	-	-	1450	-
67		Nocardiopsaceae	*Nocardiopsis sp.* strain TXV7-8SG2	7	-	65000	-	-	1600	-
68	Wu *et al*. (2007)	Acidithiobacillaceae	*Acidithiobacillus ferrooxidans* strain 16	2	-	-	32000	-	3500	-
69		Acidithiobacillaceae	*Acidithiobacillus ferrooxidans* strain H1	2	-	-	19000	-	400	-

N	Author/s	Family	*Species*	pH	Cd	Cr	Cu	Fe	Pb	Zn
70	Bafana *et al.* (2015)	Bacillaceae	*Lysinibacillus sphaericus* strain G1	7	21	49	-	-	-	23
71	Abbaszade *et al.* (2020)	Burkholderiaceae	*Cupriavidus campinensis*	7	-	-	-	-	1860	-

Table S4. Metal-remediating bacteria reported in the analyzed studies, including experimentally measured remediation concentrations ($mg\cdot kg^{-1}$) and the pH conditions under which remediation occurred.

N	Author/s	Family	Species	Method	pH	Cd	Cr	Cu	Fe	Pb	Zn
1	Hulshof *et al.* (2003)	*Hydrogenophilaceae*	*T. thioparus*	Bioleaching	7.2	4	-	1	190	-	65
2		*Acidithiobacillaceae*	*A. thiooxidans*		7.2	4	-	1	190	-	65
3			*A. ferrooxidans*		7.2	4	-	1	190	-	65
4	Han *et al.* (2023)	*Bacillaceae*	*B. megaterium*	Precipitation	3	-	-	-	-	250	-
5			*B. subtilis*		3	-	-	-	-	250	-
6			*B. megaterium*		5	-	-	-	-	305	-
7			*B. subtilis*		5	-	-	-	-	435	-
8			*B. megaterium*		9	-	-	-	-	420.5	-
9			*B. subtilis*		9	-	-	-	-	495	-
10	Chang *et al.* (2019)	*Pseudomonaceae*	*P. sp.* strain DC-B3	Biosorption	2	-	32	-	-	-	-
11	Oyetibo *et al.* (2021)	*Moraxellaceae*	*A. pittii*	Precipitation	8.2	139.3	-	-	-	593.3	-
12		*Planococcaceae*	*S. koreensis*		8.2	139.3	-	-	-	593.3	-
13		*Enterobacteriaceae*	-		8.2	139.3	-	-	-	593.3	-
14		*Bacillaceae*	*B. cereus*		8.2	139.3	-	-	-	593.3	-
15		*Exiguobacteriaceae*	*E. aurantiacum*		8.2	139.3	-	-	-	593.3	-
16		*Pseudomonaceae*	*P. citronellolis*		8.2	139.3	-	-	-	593.3	-
17	Zhu *et al.* (2019)	*Enterobacteriaceae*	*S. marcescens*	Immobilization	5.6	-	-	-	-	4	-
18					5.6	-	-	-	-	6	-
19					5.6	-	-	-	-	36	-
20					6.8	-	-	-	-	20	-
21					6.8	-	-	-	-	66	-
22					6.8	-	-	-	-	156	-
23					5.6	0.04	-	-	-	-	-
24					5.6	0.25	-	-	-	-	-
25					5.6	1.8	-	-	-	-	-
26					6.8	0.17	-	-	-	-	-
27					6.8	1.15	-	-	-	-	-
28					6.8	4	-	-	-	-	-
29					5.6	-	-	6	-	-	-
30					5.6	-	-	20	-	-	-
31					5.6	-	-	28	-	-	-
32					6.8	-	-	8.5	-	-	-
33					6.8	-	-	34	-	-	-

N	Author/s	Family	Species	Method	pH	Cd	Cr	Cu	Fe	Pb	Zn
34					6.8	-	-	40	-	-	-
35	Groudev *et al.* (2001)	*Hydrogenophilaceae*	*T. thioparus*	Immobilization	7.6	2.4	-	260	-	123	-
36			*T. neapolitanus*		7.6	2.4	-	260	-	123	-
37			*T. denitrificans*		7.6	2.4	-	260	-	123	-
38			*T. novellus*		7.6	2.4	-	260	-	123	-
39	Liu *et al.* (2021)	*Pseudomonaceae*	*P. putida*	Biosorption	7.6	3	9	-	-	10	-
40	Zheng *et al.* (2023)	*Enterobacteriaceae*	*S. marcescens* strain WZ14	Biosorption	6.95	35	-	-	-	111	-
41			*S. marcescens* strain WZ14		6.96	21.5	-	-	-	79	-
42	Sur *et al.* (2022)	*Hydrogenophilaceae*	*T. ferrooxidans*	Bioleaching	5.35	-	63	816.1		643.8	-
43	Pruden *et al.* (2007)	*Desulfobacteriaceae*	-	Bioleaching	6	0.24	-	-	-	-	0.43
44	Ghosh *et al.* (2025)	*Bacillaceae*	*Cytobacillus firmus* strain BS4	Biosorption	7	19	-	-	-	-	-
45		*Paenibacillaceae*	*Paenibacillus massiliensis* strain BS10		7	16	-	-	-	-	-
46	Kang *et al.* (2015)	*Enterobacteriaceae*	*Enterobacter cloacae* strain KJ-46	Precipitation	7	-	-	-	-	4.9 mg/L	-
47			*Enterobacter cloacae* strain KJ-47		7	-	-	-	-	3.2 mg/L	-
48	Yang *et al.* 2025	*Enterobacteriaceae*	*Enterobacter sp.* strain SX4	Precipitation	7	0.9	-	-	-	-	-
49	Liu *et al.* (2022)	*Pseudomonaceae*	*Pseudomonas taiwanensis* strain ZM11	Precipitation/ Biosorption	6.5-7.0	12.5–20	-	30–45	-	45–75	16–28
50	Hu *et al.* (2024)	*Bacillaceae*	*Lysinibacillus capsici* strain TSBLM	Precipitation	5.16	-	-	18	-	28.8	-
51	Nicolova *et al.* (2017)	*Acidithiobacillaceae*	*Acidithiobacillus ferrooxidans*	Bioleaching/Precipitation	4.39	-	-	140	-	-	-
52			*Acidithiobacillus ferrooxidans*		2.8	-	-	-	-	-	168
53		*Leptospirillaceae*	*Leptospirillum ferrooxidans*		3	-	-	140	-	-	-
54		*Desulfitobacteriaceae*	*Desulfosporosinus acidianus*		3	4.4	-	-	-	-	-
